# Modified Criteria for Radiographic Response Assessment in Glioblastoma Clinical Trials

**DOI:** 10.1007/s13311-016-0507-6

**Published:** 2017-01-20

**Authors:** Benjamin M. Ellingson, Patrick Y. Wen, Timothy F. Cloughesy

**Affiliations:** 1 UCLA Brain Tumor Imaging Laboratory, Center for Computer Vision and Imaging Biomarkers, University of California Los Angeles, 924 Westwood Blvd., Suite 615, Los Angeles, CA 90024 USA; 2 Department of Radiological Sciences, University of California Los Angeles, Los Angeles, CA USA; 3 Department of Psychiatry and Biobehavioral Sciences, David Geffen School of Medicine, University of California Los Angeles, Los Angeles, CA USA; 4 Center for Neuro-Oncology, Dana-Farber/Brigham and Women’s Cancer Center, Harvard Medical School, Boston, MA USA; 5 UCLA Neuro-Oncology Program, University of California Los Angeles, Los Angeles, CA USA; 6 Department of Neurology, David Geffen School of Medicine, University of California Los Angeles, Los Angeles, CA USA

**Keywords:** Glioblastoma, GBM, Response Assessment, T1 Subtraction, RANO

## Abstract

Radiographic endpoints including response and progression are important for the evaluation of new glioblastoma therapies. The current RANO criteria was developed to overcome many of the challenges identified with previous guidelines for response assessment, however, significant challenges and limitations remain. The current recommendations build on the strengths of the current RANO criteria, while addressing many of these limitations. Modifications to the current RANO criteria include suggestions for volumetric response evaluation, use contrast enhanced T1 subtraction maps to increase lesion conspicuity, removal of qualitative non-enhancing tumor assessment requirements, use of the post-radiation time point as the baseline for newly diagnosed glioblastoma response assessment, and “treatment-agnostic” response assessment rubrics for identifying pseudoprogression, pseudoresponse, and a confirmed durable response in newly diagnosed and recurrent glioblastoma trials.

**Electronic supplementary material:**

The online version of this article (doi:10.1007/s13311-016-0507-6) contains supplementary material, which is available to authorized users.

## Introduction

Approximately 89,000 new primary brain tumors are diagnosed in the United States each year, for which 27% are gliomas and 32.8% are malignant [1]. Glioblastoma (GBM) occurs in approximately 46% of gliomas [1] and has a poor prognosis of around 14 months median survival [2] and less than 10% of patients live longer than 5 years from diagnosis [3]. The current standard of care for newly diagnosed GBM patients consists of maximum safe surgical resection followed by external beam radiation therapy plus concomitant and adjuvant temozolomide [2], particularly in patients that demonstrate O6-methylguanine-methyltransferase (MGMT) promoter methylation. At recurrence there is no consensus as to the standard of care as no therapeutic options have produced substantial survival benefit [4].

Although overall survival (OS) is the standard for determining GBM treatment efficacy, using OS as an endpoint when studying new therapeutic strategies can be problematic because of potential influence of therapies prior to or subsequently following the therapy being studied. For example, it is difficult to definitively conclude that bevacizumab has no efficacy in GBM when a large percentage of patients in the placebo arms in both III trials studying efficacy of bevacizumab (i.e. AVAglio and RTOG-0825) eventually crossed over and received bevacizumab (31% in AVAglio [5] and 48% in RTOG-0825 [6]). If bevacizumab increased OS when given at any time during treatment, we may expect both treatment arms to have similar median OS since most patients eventually were treated with bevacizumab, disguising any therapeutic effects of the drug. Together, these results suggest OS may not be a suitable endpoint when studying new therapeutics or when there is a high chance of cross over in the control arm.

To overcome the limitations associated with using OS as the primary endpoint in studies involving new therapeutics, progression-free survival (PFS) and objective response rate (ORR) should be considered important end points [7]. However, PFS and ORR also have challenges, as determination of response and progression using anatomic imaging techniques may suffer from issues associated with measurement variability and discordance in interpretation between radiologists [8]. Therefore, it is important to develop both new response guidelines for identifying these issues as well as new imaging tools for better differentiating treatment-related changes from changes associated with non-responsive, growing tumor.

The goal of this modified response criteria is to meaningfully evaluate radiographic response and progression while simultaneously allowing therapies that may have transient effects on contrast enhancement but therapeutic benefit to be treated equally. This is particularly important in the context of platform trials, where many different therapies may be compared against a common control and there is a significant risk of over or under estimating tumor burden with a single evaluation time point. By allowing patients to stay on therapy longer, a more comprehensive and accurate assessment of therapeutic benefit can be performed on retrospective examination. A universal set of principles and guidelines, rather than treatment-specific response criteria, may allow us to fully understand the possible therapeutic benefits and potential limitations of promising new therapies for patients with GBM.

## Brief History of Radiologic Response Assessment in GBM

The formation of new blood vessels, or angiogenesis, is critical for the growth of malignant brain tumors [9–11]. Malignant gliomas with high neovascularity or vascular permeability [12–14] are often associated with higher proliferation rates [15] and higher degree of aggressivity. Because of this association, imaging techniques aimed at identifying abnormal vascularity or vascular permeability, including contrast-enhanced computed tomography (CT) and magnetic resonance imaging (MRI) are commonly used for diagnosis and clinical management of brain tumors, as they have been shown to contain the most aggressive portions of the tumor [16, 17].

In 1990, Macdonald et al. [18] introduced the first radiographic response assessment specific to brain tumors by significantly improving upon the Levin criteria [19] and the WHO oncology response criteria [20]. By standardizing the definition of radiographic response using quantitative bidirectional measurements and accounting for corticosteroid use in neurological status, similar to the response evaluation criteria in solid tumors (RECIST) [21], the new “Macdonald criteria” utilized measurements of contrast enhancing tumor size combined with other clinical metrics to determine treatment response and tumor progression by stratifying response into four categories: complete response (CR), partial response (PR), stable disease (SD), and progressive disease (PD). The original Macdonald criteria continues to be the fundamental framework for response assessment and radiographic interpretation of treatment changes in neuro-oncology, having been used for more than 20 years.

## Known Limitations for Current Response Criteria

Although contrast enhancement has been used to assess brain tumor response for more than 60 years and contrast enhancement is generally a strong surrogate of brain tumor disease, there are caveats and exceptions that have been discovered as a result of different treatment mechanisms that affect vascular permeability. For example, increased vascular permeability from cytotoxic therapies including radiotherapy and anti-neoplastic treatments have been shown to result in increased contrast enhancement in the context of therapeutic benefit, a phenomena known as “pseudoprogression.” Additionally, clinical studies examining the efficacy of new anti-angiogenic agents have noticed a substantial decrease in contrast enhancement [22–31] resulting in high response rates, ranging from 28 to 63% in bevacizumab [32–34] and 50% in cediranib [31] compared with < 10% using other chemotherapies [35–38], which translated into prolonged PFS but no difference in OS [31, 32]. It was assumed this high response rate was due to the use of contrast enhancement as the primary tool for evaluation in the Macdonald criteria, which resulted in a “pseudoresponse”[39], where contrast enhancement is falsely reduced due to changes in vascular permeability independent of anti-tumor effect.

In addition to increased response rates, studies examining tumor relapse/progression while on anti-angiogenic agents note a tendency for growth of nonenhancing, infiltrative tumor prior to emergence of contrast enhancement [25]. Approximately 30-40% of patients are estimated to experience non-enhancing tumor progression prior to changes in contrast enhancement [40, 41]. Malignant gliomas are known to contain proportions of both neovascularized and infiltrative tumor [42, 43] and the relative proportions are thought to reflect different biological phenotypes [44–48]. In 2010, expert opinion and examination of these limitations resulted in the creation of a formal Response Assessment in Neuro-Oncology (RANO) criteria [49] to comprehensively reform the Macdonald criteria using previously documented perspectives and approaches [50–52].

Although the RANO criteria corrects for a number of insufficiencies identified in the Macdonald criteria including inclusion of the evaluation of nonenhancing tumor progression and issues associated with pseudoresponse and pseudoprogression, there remain significant limitations to the current standard RANO criteria given recent data. For example, the current RANO criteria requires use of bidirectional measurements of contrast enhancing tumor size, which have been shown to overestimate tumor volume [53] and result in higher reader discordance [8, 54–59], presumably due to differences in head tilt and accurate identification of longest and perpendicular diameter in relatively irregular tumors. Other studies have shown reasonable agreement between bidimensional and volumetric measurements [60, 61], suggesting quick bidimensional assessment of contrast enhancing tumor size may be a practical alternative to more sophisticated volumetric segmentation. Additionally, the thresholds used to define response and progression is relatively arbitrary and not optimized based on scientific data showing the best correlation with survival benefit or time to treatment failure. (Note: The efficacy of these thresholds remains to be sufficiently challenged). Also, the use of thresholds based on “percentage change” with respect to baseline tumor size are significantly biased toward small tumors where relatively low *absolute* changes in tumor size are interpreted as a large *percentage* change [61]. This is particularly an issue in newly diagnosed GBM studies, where patients with tiny tumors often progress early due to triggering of progression (PD) when “non-measurable disease”, defined as having the two largest perpendicular diameters of a contrast enhancing target lesion less than 10mm, reaches the subtle threshold of “measurable disease”. Lastly, although changes in non-enhancing disease were added to the RANO criteria in an attempt to identify non-enhancing tumor progression, particularly in the presence of anti-angiogenic therapy, retrospective evaluations in clinical trials have shown it results in PD approximately a month prior to contrast enhancing disease progression [62], does not result in significant differences in prediction of OS [62, 63], and is one of the most controversial aspects of RANO evaluation due to the subjective nature of the interpretation and high adjudication rates. Further, studies have shown that specific aspects of non-enhancing tumor progression (e.g. circumscribed vs. infiltrative T2 changes) result in dramatically different post-progression survival in GBM patients [41], suggesting evaluation of non-enhancing tumor progression using T2 and/or FLAIR may be more complex than once thought and warrant further investigation before it can be properly integrated as an early radiographic endpoint. Further, new immunotherapy agents can also cause inflammation leading to changes in T2 signal intensity that is ambiguous with regard to interpretation of changes in tumor biology.

## Updated Strategies for Response Assessment in Neuro-Oncology: Modified RANO Criteria

Based on these various challenges, an update to the current response criteria is necessary in an attempt to establish a general framework for response assessment in neuro-oncology that is agnostic to the mechanism of action of the particular therapy (e.g. anti-angiogenic, anti-neoplastic, immunotherapy, etc.), each of which has its own challenges associated with interpretation of radiographic changes, and is updated based on recent scientific evidence and current clinical convention. In order to advance the RANO criteria and address these challenges we propose the following “modified” RANO criteria for use in evaluating therapeutic efficacy in patients with GBM.

### Image Acquisition Requirements

In response to a need for better standardization of image acquisition in GBM clinical trials [64], a recent consensus paper was published outlining an “international brain tumor imaging protocol (BTIP)” (Table 1) with recommended sequences and parameters [65]. At the core of this recommended protocol is parameter matched, pre- and post-contrast 3D (volumetric) inversion recovery gradient recalled echo (IR-GRE) images with less than 1.5-mm isotropic resolution, which allows for both bidimensional and volumetric measurements of enhancing tumor. When possible, this protocol should be employed for prospective clinical trials.

**Table 1:** International Standardized Brain Tumor Imaging Protocol (BTIP) minimum image acquisition requirements for 1.5T and 3T MR systems

Variable	3D T1w Pre^b^	Ax 2D FLAIR^j^	Ax 2D DWI	Contrast Injection^a^	Ax 2D T2w^h,i^	3D T1w Post^b^
Sequence	IR-GRE^e,f^	TSE^c^	SS-EPI^g^		TSE^c^	IR-GRE^e,f^
Plane	Sagittal/Axial	Axial	Axial		Axial	Sagittal/Axial
Mode	3D	2D	2D		2D	3D
TR [ms]	2100^m^	>6000	>5000		>2500	2100^m^
TE [ms]	Min	100-140	Min		80-120	Min
TI [ms]	1100^n^	2000-2500^k^				1100^n^
Flip angle	10°-15°	90°/≥160°	90°/180°		90°/≥160°	10°-15°
Frequency	≥172	≥256	≥128		≥256	≥172
Phase	≥172	≥256	≥128		≥256	≥172
NEX	≥1	≥1	≥1		≥1	≥1
FOV	256mm	240mm	240mm		240mm	256mm
Slice thickness	≤1.5mm	≤4mm^l^	≤4mm^l^		≤4mm^l^	≤1.5mm
Gap/Spacing	0	0	0		0	0
Diffusion options^p^			*b* = 0, 500, 1000 s/mm^2^ ≥3 directions			
Parallel imaging	Up to 2x	Up to 2x	Up to 2x		Up to 2x	Up to 2x
Approximate scan time	5-10 min	4-8 min	2-4 min		4-8 min	5-10 min

Ax = Axial; ADC = apparent diffusion coefficient; FLAIR = fluid attenuated inversion recovery; DWI = diffusion-weighted imaging; 3D = three dimensional; TSE = turbo spin echo; EPI = echo planar imaging; SS-EPI = single-shot echo planar imaging; GE-EPI = gradient echo echo planar imaging; 2DFL = two-dimensional FLASH (fast low angle shot) gradient recalled echo; MPRAGE = magnetization prepared rapid gradient-echo; A/P = anterior to posterior; R/L = right to left; NEX = number of excitations or averages; FOV = field of view; TE = echo time; TR = repetition time; TI = inversion time; PD = proton density; DSC = dynamic susceptibility contrast; IR-GRE = inversion-recovery gradient-recalled echo

^a^0.1 mmol/kg dose injection with a Gadolinium chelated contrast agent. Use of a power injector is desirable at an injection rate of 3-5cc/s

^b^Post-contrast 3D T1-weighted images should be collected with equivalent parameters to pre-contrast 3D T1-weighted images

^c^TSE = turbo spin echo (Siemens & Philips) is equivalent to FSE (fast spin echo; GE, Hitachi, Toshiba)

^d^FL2D = two-dimensional fast low angle shot (FLASH; Siemens) is equivalent to the spoil gradient recalled echo (SPGR; GE) or T1- fast field echo (FFE; Philips), fast field echo (FastFE; Toshiba), or the radiofrequency spoiled steady state acquisition rewound gradient echo (RSSG; Hitachi). A fast gradient echo sequence without inversion preparation is desired

^e^IR-GRE = inversion-recovery gradient-recalled echo sequence is equivalent to MPRAGE = magnetization prepared rapid gradient-echo (Siemens & Hitachi) and the inversion recovery spoiled gradient-echo (IR-SPGR or Fast SPGR with inversion activated or BRAVO; GE), 3D turbo field echo (TFE; Philips), or 3D fast field echo (3D Fast FE; Toshiba)

^f^A 3D acquisition without inversion preparation will result in different contrast compared with MPRAGE or another IR-prepped 3D T1-weighted sequences and therefore should be avoided

^g^In the event of significant patient motion, a radial acquisition scheme may be used (e.g. BLADE [Siemens], PROPELLER [GE], MultiVane [Philips], RADAR [Hitachi], or JET [Toshiba]); however, this acquisition scheme is can cause significant differences in ADC quantification and therefore should be used only if EPI is not an option. Further, this type of acquisition takes considerably more time

^h^Dual echo PD/T2 TSE is optional for possible quantification of tissue T2. For this sequence, the PD echo is recommended to have a TE < 25ms

^i^Advanced sequences can be substituted into this time slot, so long as 3D post-contrast T1-weighted images are collected between 4 and 8 min after contrast injection

^j^3D FLAIR is an optional alternative to 2D FLAIR, with sequence parameters as follows per EORTC guidelines: 3D TSE/FSE acquisition; TE = 90-140ms; TR = 6000-10000ms; TI = 2000-2500ms (chosen based on vendor recommendations for optimized protocol and field strength); GRAPPA ≤ 2; Fat Saturation; Slice thickness ≤ 1.5mm; Orientation Sagittal or Axial; FOV ≤ 250 mm x 250 mm; Matrix ≥ 244x244

^k^Choice of TI should be chosen based on the magnetic field strength of the system (e.g. TI ≈ 2000ms for 1.5T and TI ≈ 2500ms for 3T)

^l^In order to ensure comparable SNR older 1.5T MR systems can use contiguous (no interslice gap) images with 5mm slice thickness or increase NEX for slice thickness ≤4mm

^n^For Siemens and Hitachi scanners. GE, Philips, and Toshiba scanners should use a TI = 400-450ms for similar contrast

^m^For Siemens and Hitachi scanners. GE, Philips, and Toshiba scanners should use a TR = 5-15ms for similar contrast

^p^Older model MR scanners that are not capable of >2 *b*-values should use *b* = 0 and 1000 s/mm^2^

If volumetric acquisition is not employed, or if retrospective evaluations of existing trial data are performed, then slice thickness plus interslice gap should be less than 5 mm. If the sum of the slice thickness and gap exceeds 5 mm, then slightly modified definitions of measurable disease should be used (e.g. measurable disease = largest perpendicular diameters > 2× slice thickness + gap).

### Contrast Enhanced T_1_-Weighted Digital Subtraction Maps for Increased Lesion Conspicuity

**Fig. 1:**
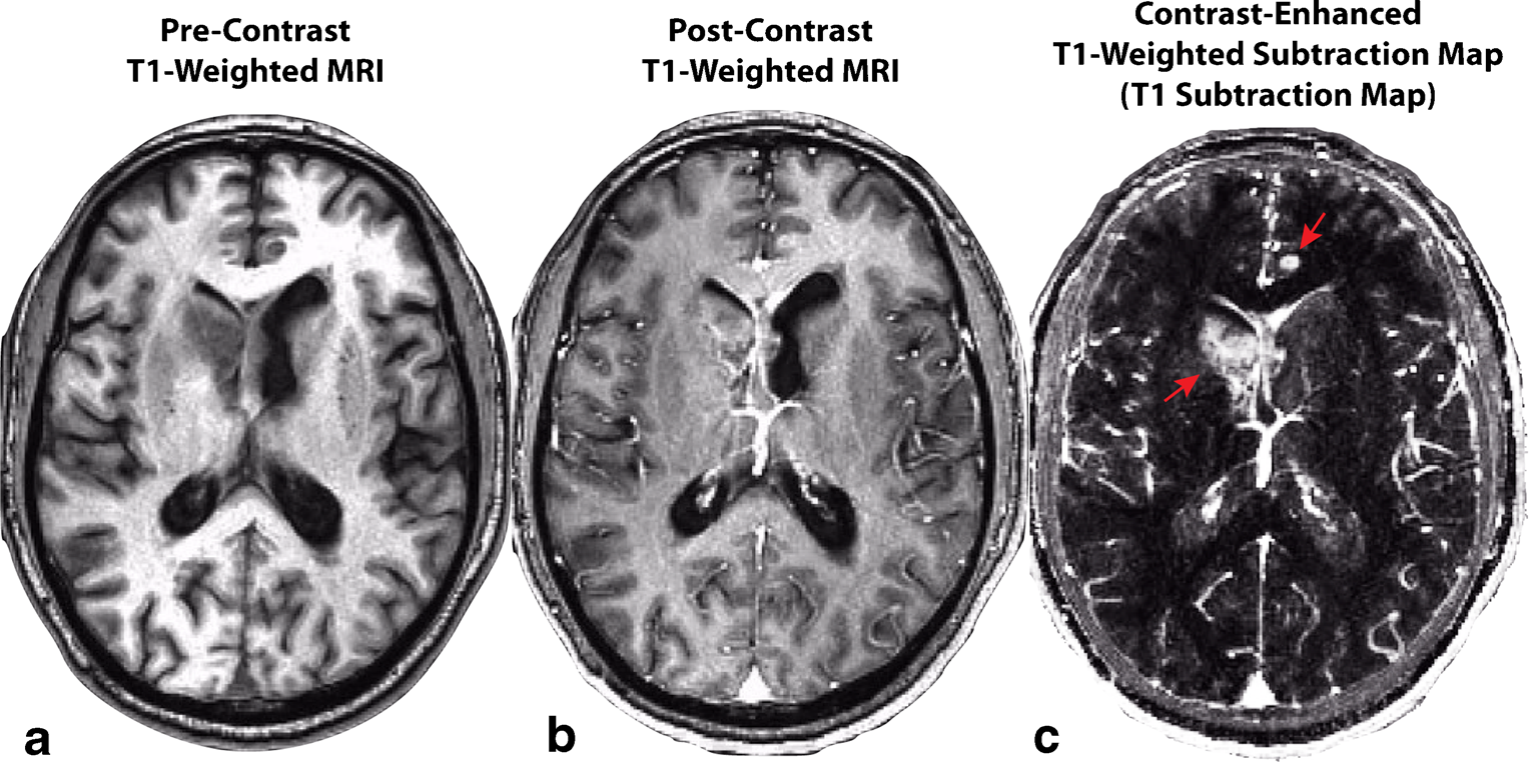
Construction of contrast enhanced T1-weighted subtraction maps in a recurrent glioblastoma patient treated with bevacizumab. A) Pre-contrast T1-weighted MR image. B) Post-contrast T1-weighted MR image. C) T1 subtraction map calculated by voxel-wise subtraction of pre-contrast from post-contrast T1-weighted images highlighting areas of increased contrast enhancement. *Red arrows* show two subtly enhancing lesions that are easily identified on T1 subtraction maps

Quantification of contrast enhancing tumor size or volume should be performed on contrast-enhanced T_1_-weighted digital subtraction maps (Fig. 1) in order to increase lesion conspicuity and better predict tumor burden in the presence of reduced vascular permeability as occurs during anti-angiogenic therapy [66] and/or T_1_ shortening from blood products or calcifications [67, 68]. Further, the American College of Radiology (ACR) recommends this approach for identification and delineation of subtly enhancing bone and soft tissue lesions [69].

### Bidimensional and/or Volumetric Measurements

Similar to the current RANO criteria, two-dimensional, perpendicular measurements of contrast enhancing tumor size, excluding the resection cavity along with any cysts or areas of central macroscopic necrosis, should be used for response assessment if volumetric tools are not available. Table 2 outlines suggested volumetric conversions from two- to three-dimensional measurements for consistency in response definitions, as outlined by Chappell et al. [70].

**Table 2:** Bidimensional to volumetric definitions [54, 70, 96] of radiographic response and progression

State of disease	Change in bidimensional product	Estimated volumetric change
Complete response (CR)	100% Decrease	100% Decrease
Partial response (PR)	≥50% Decrease	≥65% Decrease
Progressive disease (PD)	≥25% Increase	≥40% Increase
Stable disease (SD)	<50% Decrease to <25% Increase	<65% Decrease to <40% Increase

It is important to note that the field remains conflicted on whether or not *enhancing disease* should be included in tumor size measurements, or whether it is more appropriate to monitor *total enhancing lesion volume*, which may include central macroscopic necrosis and any cystic components (but excluding surgically resected tissue). Scientific studies have shown that *both* approaches for quantifying change in tumor size as a surrogate of treatment response are valuable. Multiple studies utilizing the Macdonald and RANO criteria have shown that change in enhancing disease size using bidimensional measurements, excluding necrosis and cystic components, can be used to predict survival in a variety of therapies. A recent study from the BRAIN trial, a phase II trial of bevacizumab with or without irinotecan in recurrent GBM, confirmed that change in the volume of enhancing disease can be used to predict survival benefit [66]. However, a recent study examining growth rates in treatment naïve presurgical GBMs showed that changes in enhancing disease only may not be reliable, since changes occurring prior to any therapy often showed stable or decreasing tumor enhancing disease volume [61]. Growth rates were universally positive (i.e. growing) when total lesion volume (including central necrosis) were taken into consideration, which appears more realistic given the fast growth trajectory of these tumors during therapeutic intervention. Regardless, future studies are warranted to determine which measurement may be more clinically meaningful or reliable in predicting early response to new therapies.

### Definition of Measurable Disease, Non-Measurable Disease, and Target Lesions

**Fig. 2:**
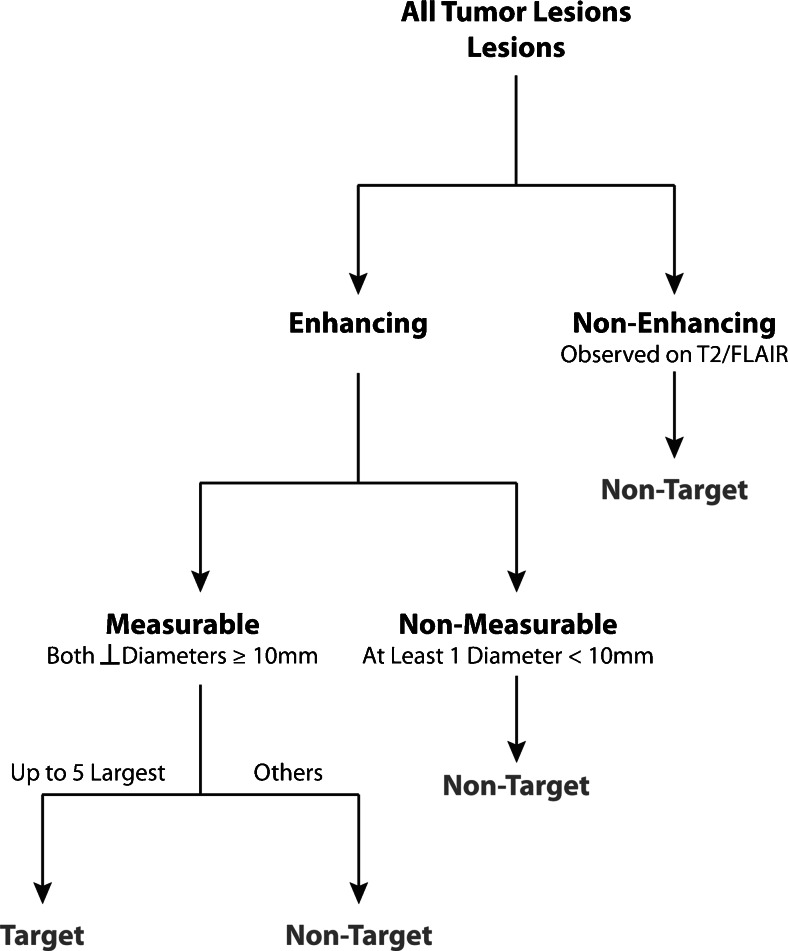
Algorithm for identifying measurable and target lesions

Measurable disease should be defined as contrast enhancing lesions with a minimum size of *both* perpendicular measurements greater than or equal to 10mm (Fig. 2). For example, if the largest diameter is 15 mm but the perpendicular diameter is 8 mm, this would constitute *non*-*measurable disease*. Additionally, in the event that the BTIP protocol is not used, if the slice thickness plus interslice gap is greater than 5mm, then the minimum size for both perpendicular measurements should be twice the sum of the slice thickness and interslice gap (e.g. if the slice thickness is 5mm with 1.5mm interslice gap, the minimum tumor size on both perpendicular dimensions should be 13 mm). Up to a total of five target measurable lesions should be defined and ranked from largest to smallest (Fig. 2).

Non-measurable disease should be defined as lesions that are too small to be measured (less than 1 cm in both perpendicular dimensions), lesions that lack contrast enhancement (non-enhancing disease), or lesions that contain a poorly defined margin that cannot be measured or segmented with confidence.

### Correction for “Baseline Tumor Volume” in Newly Diagnosed and Recurrent GBM

An abundance of single center, multicenter, and phase I-III trials have confirmed that baseline contrast enhancing tumor size (volume or bidirectional measurements) is a significant prognostic factor contributing to overall survival (OS) in GBM. In newly diagnosed GBM, both extent of resection [3, 71–87] and post-surgical residual volume [83–85, 88–92] have been shown to be prognostic. Similarly, baseline pre-treatment contrast enhanced tumor size has also been shown to be prognostic for OS in recurrent GBM [53, 66, 93]. However, from a clinical trial perspective, post-surgical residual enhancing tumor volume may be a more practical measurement to obtain, as pre-surgical MRI scans are often not available or collected as part of clinical trials because patients are not enrolled until after surgery and diagnosis. Thus, care should be made to make sure baseline tumor size is a stratification factor during randomization (i.e. prospectively balanced across treatment arms) and used as a covariate in statistical models evaluating treatment efficacy.

### Post-Radiation MRI Examination as the Reference for Evaluating Radiographic Response in Newly Diagnosed GBM

The current RANO criterion defines the post-surgical MRI scan as the baseline for treatment response evaluation; however, we propose using the post-radiation examination (i.e. the first scan following completion of concurrent radiation therapy and chemotherapies such as temozolomide and/or experimental therapeutics) as the baseline for response assessment because reliability of tumor assessment on the post-surgical MR scans can be problematic for a number of reasons. First, this scan is typically acquired prior to a final pathological diagnosis, thus patients are not yet enrolled in a clinical trial and therefore the imaging protocol may not be consistent with trial recommendations, leading to a mismatch between the baseline and subsequent follow-up time points. Secondly, post-operative MR scans are often contaminated with post-surgical changes including blood products and increased vascular permeability from surgical trauma. Thirdly, steroid dose can be highly variable during this time and may be poorly annotated, as patients are typically not yet enrolled in clinical trials at this point. Additionally, the timing of the post-operative MR scans can be highly variable from patient to patient, depending on the complexity of the surgery and potential intraoperative complications, and institution by institution, as many factors including availability of inpatient MR scanners can lead to different timing of the post-surgical MRI evaluation. This variability inevitably leads to differing degrees of post-surgical artifacts and fluid levels on the resulting images. Together, these factors appear to indicate the post-surgical MRI examination may not be a reliable reference scan for accurately determining radiographic changes, despite post-surgical residual enhancing volume being a significant prognostic factor as outlined above.

Perhaps the most compelling argument for using the post-radiation scan as the baseline for determining response assessment is the highly unpredictable, transient radiographic changes that often accompany the initial chemoradiation phase (i.e. external beam radiation therapy plus concurrent temozolomide) with or without experimental therapeutics. Within 1 month after completion of standard chemoradiation therapy, approximately 50% of patients will experience radiographic changes suggestive of early tumor progression in reference to the post-surgical MRI exam, of which 50% are likely to have pseudoprogression (i.e. 25% of all patients at 1 month post-chemoradiation are estimated to have pseudoprogression) [94]. This proportion of patients with both early progression and pseudoprogression decreases steadily during the subsequent standard adjuvant chemotherapy phase, which forms the basis for current RANO recommendations of excluding patients in recurrent GBM trials who progressed within 3 months after completion of chemoradiation. Many clinicians are reluctant to change therapy based on this examination due to the relatively high incidence of treatment-related radiographic changes directly after completion of concurrent chemotherapy and radiation, and instead use this scan as a new baseline in which to interpret subsequent changes in tumor size. Additionally, experimental therapeutics that significantly alter vascular permeability, including anti-angiogenic and immunotherapies, when used concurrently with radiation therapy and temozolomide often demonstrate dramatic and transient changes in contrast enhancement that quickly stabilize following completion of radiation [95]. Despite the improved lesion conspicuity on T1 subtraction maps in the settings of these therapies, these early changes between the post-surgical, pre-radiation exam and the post-radiation exam may not accurately reflect true changes in tumor burden nor predict long-term survival benefit [95].

## Detailed Definitions Used for Modified Radiographic Response Assessment Criteria

Radiographic response should be determined in comparison to the tumor measurements obtained at baseline (post-radiation scan will be baseline for newly diagnosed GBM and pre-treatment scans will be the baseline for recurrent GBM) for determination of response, and the smallest tumor measurement at either pre-treatment baseline or following initiation of therapy for determining progression.

Because novel treatments are likely to result in a higher than normal incidence of treatment-related increase in contrast enhancement (“pseudoprogression”, PsP) or decrease in contrast enhancement (“pseudoresponse”, PsR), patients should continue therapy with close observation (e.g. 4-8 week intervals) if there is a suspicion of PsP or PsR. If subsequent imaging studies and/or clinical observations demonstrate that progression in fact has occurred, the date of confirmed progression should be noted as the scan at which the potential progression was first identified. Definitions for complete response, partial response, progressive disease, and stable disease should be defined as follows for all target lesions.

*Complete Response* (*CR*): Requires *all* of the following:

1. Disappearance of all enhancing measurable and non-measurable disease sustained for at least 4 weeks. The first scan exhibiting disappearance of all enhancing measurable and non-measurable disease is considered “preliminary CR”. If the second scan exhibits measurable enhancing disease with respect to the “preliminary CR” scan, then the response is not sustained, noted as pseudoresponse, PsR, and is now considered “preliminary PD” (note confirmed PD requires at least two sequential increases in tumor volume). If the second scan continues to exhibit disappearance of enhancing disease or emergence of non-measurable disease (less than 10mm bidimensional product), it is considered a *durable CR* and the patient should continue on therapy until confirmed PD is observed.
2. Patients must be off corticosteroids (or on physiologic replacement doses only).
3. Stable or improved clinical assessments (i.e. neurological examinations).

Note: Patients with non-measurable disease only at baseline cannot have CR; the best response possible is stable disease (SD).

*Partial Response* (*PR*): Requires *all* of the following:

1. ≥50% decrease in sum of products of perpendicular diameters or ≥65% decrease in total volume [54, 70, 96] of all measurable enhancing lesions compared with baseline, sustained for at least 4 weeks. The first scan exhibiting ≥50% decrease in sum of products of perpendicular diameters or ≥65% decrease in total volume [54, 70, 96] of all measurable enhancing lesions compared with baseline is considered “preliminary PR”. If the second scan exhibits PD with respect to the “preliminary PR” scan, then the response is not sustained, noted as pseudoresponse, PsR, and is now considered “preliminary PD” (note confirmed PD requires at least two sequential increases in tumor volume). If the second scan exhibits SD, PR, or CR, it is considered a *durable PR* and the patient should continue on therapy until confirmed PD is observed.
2. Steroid dose should be the same or lower compared with baseline scan.
3. Stable or improved clinical assessments.

Note: Patients with non-measurable disease only at baseline cannot have PR; the best response possible is stable disease (SD).

*Progressive Disease* (*PD*): Defined by any of the following:

1. At least two sequential scans separated by at ≥4 weeks both exhibiting ≥25% increase in sum of products of perpendicular diameters or ≥40% increase in total volume [54, 70, 96] of enhancing lesions. The first scan exhibiting ≥25% increase in sum of products of perpendicular diameters or ≥40% increase in total volume [54, 70, 96] of enhancing lesions should be compared to the smallest tumor measurement obtained either at baseline (if no decrease) or best response (on stable or increasing steroid dose) and is noted as “preliminary PD.” If the second scan at least 4 weeks later exhibits a subsequent ≥25% increase in sum of products of perpendicular diameters or ≥40% increase in total volume of enhancing lesions relative to the “preliminary PD” scan, it is considered “confirmed PD” and the patient should discontinue therapy. If the second scan at least 4 weeks later exhibits SD or PR/CR, this scan showing “preliminary PD” is noted as “pseudoprogression”, PsP, and the patient should continue on therapy until a second increase in tumor size relative to the PsP scan is observed. Note that any new *measurable* (>10mm x 10mm) enhancing lesions should *not* be immediately considered PD, but instead should be added to the sum of bidimensional products or total volume representing the entire enhancing tumor burden.
2. In the case where the baseline or best response demonstrates no measurable enhancing disease (visible or not visible), then any new *measurable* (>10mm x 10mm) enhancing lesions are considered PD *after* confirmed by a subsequent scan ≥4 weeks exhibiting ≥25% increase in sum of products of perpendicular diameters or ≥40% increase in total volume of enhancing lesions [54, 70, 96] relative to the scan first illustrating new measurable disease. The first scan exhibiting new measurable disease is noted as “preliminary PD.” If the second scan at least 4 weeks later exhibits a subsequent ≥25% increase in sum of products of perpendicular diameters or ≥40% increase in total volume [54, 70, 96] of enhancing lesions relative to the “preliminary PD” scan it is considered “confirmed PD” and the patient should discontinue therapy. If the second scan at least 4 weeks later exhibits SD, CR, PR, or becomes non-measurable, this scan showing “preliminary PD” is noted as “pseudoprogression”, PsP, and the patient should continue on therapy until a second increase in tumor size relative to the “preliminary PD”, or PsP, scan is observed. Note that any new *measurable* (>10mm x 10mm) enhancing lesions on the subsequent scan following the preliminary PD scan should *not* be immediately considered confirmed PD, but instead should be added to the sum of bidimensional products or total volume representing the entire enhancing tumor burden.
3. Clear clinical deterioration not attributable to other causes apart from tumor (e.g. seizures, medication adverse effects, therapy complications, stroke, infection) or attributable to changes in steroid dose.
4. Failure to return for evaluation as a result of death or deteriorating condition.

*Stable Disease* (*SD*): Requires *all* of the following:

1. Does not qualify for CR, PR, or PD as defined above. Note this also applies to patients that demonstrate PsR when the confirmation scan does not show PD or PsP when the confirmation scan does not show PR/CR.
2. In the event that corticosteroid dose was increased (for new symptoms/signs) without confirmation of disease progression on neuroimaging, and subsequent follow-up imaging shows that the steroid increase was required because of disease progression, the last scan considered to show stable disease will be the scan obtained when the corticosteroid dose was equivalent to the baseline dose.

### Symptomatic Deterioration & Reporting Clinical Status

Patients with global deterioration of health status requiring discontinuation of treatment without objective evidence of disease progression at that time, and not either related to study treatment or other medical conditions, should be reported as PD due to “symptomatic deterioration.” Every effort should be made to document the objective progression even after discontinuation of treatment due to symptomatic deterioration. Neurological exam data should be provided to the independent radiologic facility as “stable, better, worse” in case report forms or from study sponsor. Clinical status should be recorded as “worse” if the neurological exam is worse, otherwise the clinical status should be set to “not worse.” In the event that necessary clinical data is not available, clinical status should be recorded as “not available” and that particular time point can only be reviewed for PD (otherwise “non-evaluable”). Neurological data must be within ±7 days of the time-point response date, otherwise the data is considered “not available”.

### Steroid Use and Dose

Steroid use should be derived from the concomitant medications on the case report forms and recorded as “Yes”, “No”, or “not available”. A value of “No” should be assigned if, at the time-point, the subject is not on steroids or on physiologic replacement doses only (<1.5 mg dexamethasone or equivalent per day).

Steroid dose should be derived from the concomitant medications on the case report forms. Average steroid dose no greater than 2 mg change from baseline should be abstracted to “stable”. If outside this range the steroid dose should be abstracted to “increased” or “decreased” accordingly. Steroid data should be within ±5 days of the time-point response date, otherwise the data is considered “not available”.

### Overall Objective Status

The overall objective status for an evaluation should be determined by combining the patient’s radiographic response on target lesions, new disease, neurological status, and steroid dose/usage as defined in Table 3 for patients with *measurable* (>10mm x 10mm) disease. Note that patients with possible PsP or pseudoresponse should be given the Objective Status of “Preliminary Progression” or “Preliminary Response”, respectively. Once PsP, pseudoresponse, or true progression/response are confirmed, the Objective Status can be changed accordingly.

**Table 3:** Guidelines for determining comprehensive objective status

Target lesions (current scan)	Target lesions (previous scan)	New sites of measurable disease^a^	Neurological status	Steroid usage	Steroid dose	Overall objective status
CR	Not Evaluated	No	Stable/Better	No	N/A	Preliminary CR
PR	Not Evaluated	No	Stable/Better	Any	Stable/Decreasing	Preliminary PR
PD	Not Evaluated	Yes or No	Stable/Better	Any	Stable/Increasing	Preliminary PD
PD	Preliminary or Confirmed PR/CR	No	Stable/Better	Any	Stable/Increasing	Preliminary PD
SD	Preliminary or Confirmed CR/PR or SD/NE	No	Stable/Better	Any	N/A	SD
PR	Preliminary PR	Yes or No	Stable/Better	Any	Stable/Decreasing	Confirmed PR
SD	Preliminary PR	Yes or No	Stable/Better	Any	Stable/Decreasing	SD (Preliminary PR →Confirmed PR)
SD	Preliminary CR	Yes or No	Stable/Better	Any	Stable/Decreasing	SD (Preliminary CR →Confirmed CR)
CR	Preliminary CR	No	Stable/Better	No	N/A	Confirmed CR
SD	Preliminary PD	No	Stable/Better	Any	Stable/Decreasing	SD (Confirmed PsP)
CR/PR/SD PD/NE	CR/PR/SD/PD/NE	Yes or No	Worse	Any	Stable/Increasing	Confirmed PD
PD	Preliminary PD	Yes or No	Any	Yes	Stable/Increasing	Confirmed PD

^a^Note that new sites of measurable disease are added to the sum of bidimensional products or total lesion volume, or constitutes preliminary PD in the case of no measurable disease at baseline or best response

## Detailed Modified Radiographic Response Assessment Rubric

In order to provide both clinical guidelines for continuing therapy *beyond* suspected radiographic progression if the treating physician believes there may be a therapeutic benefit and to provide criteria for defining progression and early drug failure while also allowing for the possibility of PsP and PsR, a modified response rubric similar to those described recently [97] should be employed. Two different rubrics should be used depending on whether the patient is newly diagnosed or enrolled in a trial for recurrent disease.

It is important to note that the primary differences between conventional RANO and the proposed modified criteria are: (1) use of the post-radiation time point as the baseline for response evaluation in newly diagnosed GBM and (2) considering only *objectively defined*, *measurable enhancing disease* in the definition of response and progression (i.e. exclusion of qualitatively assessed T2/FLAIR changes).

### Newly Diagnosed GBM (Fig. 3)

Newly diagnosed GBM patients will initially undergo a pre-entry MRI scan for initial diagnosis prior to entry in the study and prior to therapy. The post-operative scan [MRI(0)] is *desired* in order to assess residual enhancing disease volume for use as a covariate in survival analyses, as described previously. Patients will then start on standard or experimental therapy with concurrent radiation therapy (RT). The *Post*-*RT scan* [*MRI*(*1*)] *will be required and used as the baseline scan* for which response will be determined.^1^ Following the first cycles of adjuvant therapy, patients will receive additional *required* MRI scans [MRI(N)].

**Fig. 3:**
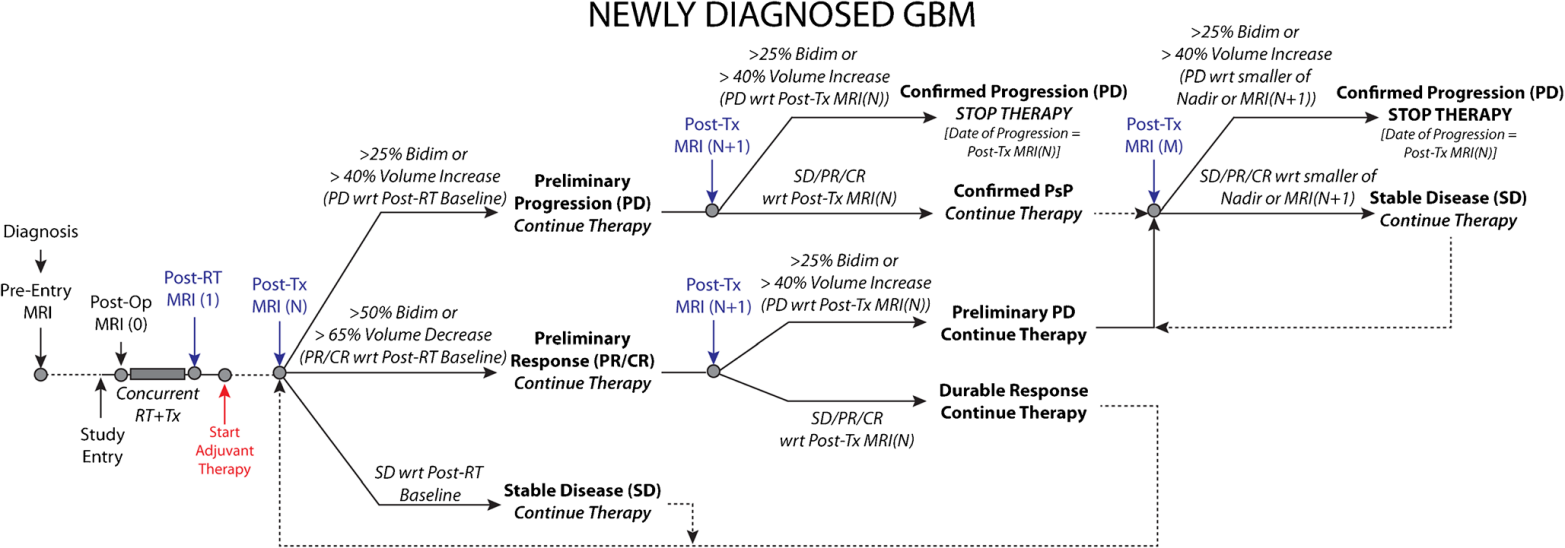
Modified radiographic response assessment rubric for management of both pseudoprogression and pseudoresponse in newly diagnosed glioblastoma

### Recurrent GBM (Fig. 4)

Recurrent GBM patients will undergo a pre-entry MRI scan [MRI(0)] at the time of recurrence. At the time of study entry, two scans to confirm progression should be submitted consisting of at least one scan at the time of progression and one scan at Nadir or baseline. If the patient undergoes surgery (optional), then the *post*-*surgical*, *pre*-*treatment* MRI can be used as the baseline [MRI(1)], assuming it is obtained < 72 hours from surgery to reduce post-operative reactive enhancement [91, 98]. (Note: If the post-operative MRI scan is used as the baseline reference, the standardized MRI protocols *must* be used.) If the patient does not go to surgery or if the start of treatment is > 21 days from the start of therapy, the patient will undergo a *pre*-*treatment* MRI [MRI(1)] scan as the baseline scan for which response will be determined. Following the first cycles of therapy, patients will receive additional MRI scans [MRI(N)].

**Fig. 4:**
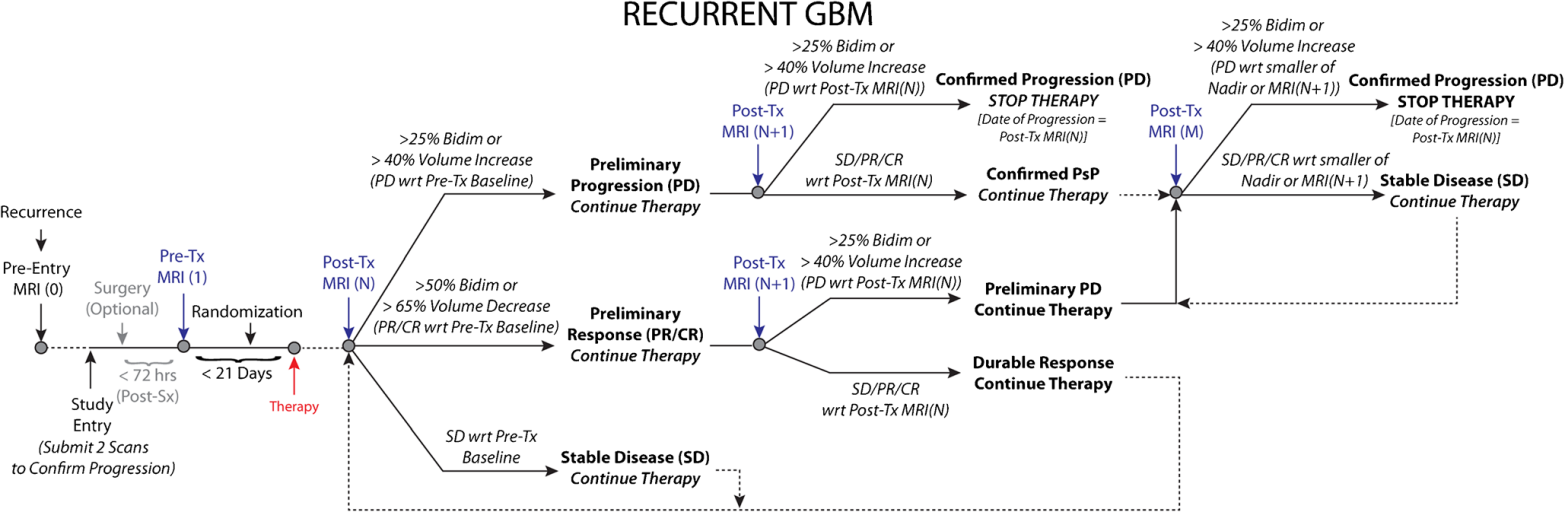
Modified radiographic response assessment rubric for recurrent glioblastoma

### Details Common to Both Newly Diagnosed and Recurrent GBM

#### Preliminary Radiographic Progression

If the lesion size has increased ≥25% sum of bidirectional product or ≥40% in volume between MRI Scan 1 and N, these patients should be categorized as “preliminary radiographic progression”. If the investigator believes the patient can safely continue on therapy, then they should continue to treat and acquire a follow-up confirmatory scan [MRI(N + 1)] at the next scan interval (8 weeks ± 4 weeks from MRI Scan (N) or no less than 4 weeks minimum duration between preliminary PD and confirmed PD scans) to verify tumor growth and progression. For patients with gross-total resection (GTR) and no measurable enhancing disease, *preliminary* radiographic progression is defined as a transition from *no measurable disease* to *non*-*measureable* (*but present*) *disease* (<*10mm x 10mm*) or *measurable disease* (>*10mm x 10mm*). If the investigator feels it is safe to keep the patient on, a confirmatory scan at MRI(N + 1) should be obtained to verify tumor progression.

#### Confirmed Progression

If the patient has an increase ≥25% sum of bidirectional product or ≥40% in volume between MRI Scan N and N + 1, this is “Confirmed Progression”, *the patient should stop therapy* and the date of radiographic progression is the date of suspected progression, MRI(N). If the patient has SD/PR/CR on MRI(N + 1) with respect to MRI(N), PsP is confirmed and the patient should continue on therapy. Patients will then continue on therapy and receive additional follow-up MRI scans [MRI(M)]. If the lesion size has increased ≥25% sum of bidirectional product or ≥40% in volume on MRI(M) relative to the smaller of Nadir or MRI(N + 1), then the patient has “Confirmed Progression”, *the patient should stop therapy* and the date of radiographic progression is the new date, MRI(M). For patients with no measurable disease at the Post-RT baseline, “Confirmed Progression” will be defined as a transition from *non*-*measurable* (*but present*) *disease* (<*10mm x* <*10mm*) on MRI(N) to *measurable disease* (>*10mm x 10mm*) on MRI(N + 1). For patients with confirmed PsP and no measurable disease at Nadir, “Confirmed Progression” should be defined as a transition from *no measurable disease* to *measurable disease* (>*10mm x 10mm*). In all cases, patients with confirmed progression should stop therapy.

#### Preliminary & Confirmed Radiographic Response

If a measurable lesion has decreased ≥50% sum of bidirectional product or ≥65% in volume between MRI(1) and MRI(N), these patients should be categorized as “preliminary radiographic responders” and will be monitored for an additional time point and/or treatment cycle. After an additional cycle of therapy (8 weeks ± 4 weeks from MRI(N)), patients will receive a confirmatory MRI(N + 1). If the lesion(s) have increased ≥25% sum of bidirectional product or ≥40% in volume from MRI(N) (indicating radiographic progression from MRI(N)), this is considered an “unsustained radiographic response” or “pseudoresponse”. The date of radiographic progression for these patients will be MRI(N + 1) and *the patient should stop therapy*. Alternatively, if the lesion has not increased from MRI(N), this is considered a “durable radiographic response,” the patient will continue on therapy, and the date of *preliminary* radiographic progression is the time point of an increase ≥25% sum of bidirectional product or ≥40% in volume (from Nadir) during the remainder of the study. The investigator can then decide whether to continue safely on therapy until progression has been confirmed and at the subsequent time point stop therapy if they feel the patient cannot safely continue therapy.

#### Stable Disease

If the lesion size has not increased or decreased beyond the set thresholds between Scan 1 and N, the patient is considered “stable.” Such patients will continue on therapy, and the date of *preliminary* progression is the time point of an increase ≥25% sum of bidirectional product or ≥40% in volume (from Nadir) during the remainder of the study. Upon preliminary progression the investigator can choose to either continue therapy and confirm progression or discontinue therapy. For cases with significant neurologic decline at the time of imaging progression as determined from MRI(N), a confirmatory scan at time point MRI(N + 1) may not be possible or necessary. For these cases, it is appropriate to define MRI(N) as the progression time point.

## Conclusions

Although radiographic response assessment is imperfect and many nuances exist, changes in contrast enhancing tumor are both clinically meaningful and appropriate for evaluating efficacy of new treatments in GBM. The outlined modifications in this report are meant to both build on the strengths of the current RANO criteria while providing potential solutions for many of the common challenges.

## Electronic supplementary material

Below is the link to the electronic supplementary material.

Required Author Forms Disclosure forms provided by the authors are available with the online version of this article. (PDF 1224 kb)

## Footnotes

1 Note the post-surgical, pre-RT scan is often corrupted with post-surgical blood products and other surgical changes. Further, it is difficult to standardize in terms of post-surgical timing, where delays in scanning often lead to increased T1 shortening on pre-contrast T1-weighted images due to blood products, etc., and therefore may not be an adequate representation of baseline tumor burden.
